# A Young Male With Anorexia, Abdominal Complaints and Marked Blood Eosinophilia

**DOI:** 10.7759/cureus.12314

**Published:** 2020-12-26

**Authors:** Dimitrios Anyfantakis

**Affiliations:** 1 Primary Care, Primary Health Care Centre of Kissamos, Chania, GRC

**Keywords:** eosinophilia, colorectal cancer

## Abstract

Peripheral blood eosinophilia represents a frequent finding in routine clinical practice when absolute eosinophil count is found to be greater than 0.5 x 109/L (500/µL). Common causes include parasitic infections, allergic reactions, and hyper-eosinophilic syndrome. Eosinophilia secondary to malignancy represents an uncommon presentation.

Here we report an atypical case of a 47-year-old previously healthy male who presented to a primary care setting complaining of fatigue and anorexia for the last two weeks. The evaluation revealed leucocytosis and peripheral hypereosinophilia with an absolute eosinophil count of 14.13×109/L (37%).

Following an extensive diagnostic work in a secondary care centre he was finally diagnosed with rectal carcinoma. This case highlights that solid malignancy should be considered in patients with marked peripheral eosinophilia.

## Introduction

Peripheral blood eosinophilia (EOS) is frequently encountered in routine clinical practice. It is defined as an increase in the absolute eosinophil count greater than 500 eosinophils/microL [1]. An accurate diagnosis of the etiology of EOS is challenging and represents a key issue for the appropriate management. In this direction, detailed history and physical examination are considered critical for the identification of the eosinophil-related organs.

Here we present an unusual case of a young man admitted with nonspecific gastrointestinal symptoms and EOS.

## Case presentation

A 47-year-old male presented to his general practitioner due to anorexia, nausea, fatigue, and alternating episodes of constipation with watery diarrhea during the last month. He was a life-long heavy smoker and a moderate alcohol consumer. His medical history was negative for medication use, allergies, skin rashes, and recent traveling.

On admission he was afebrile (36.8°C) with heart rate 85/min and blood pressure 140/80 mmHg.

Physical examination was negative for anemia, jaundice, lymphadenopathy, and organomegaly. On abdominal examination, his abdomen was distended but soft, with normal bowel sounds and no shifting dullness. The cardiac and pulmonary examination was normal. Rectal examination revealed an external hemorrhoid.

Initial laboratory work up showed leucocytosis with peripheral eosinophilia; leukocytes were 38.800 cells/mm3 (reference range: 4000-11000 per mm3) and absolute eosinophil counts 16.700 cells/mm3 (reference range: 50-500 cells/mm3). Laboratory findings were also notable for gamma-glutamyl transferase (γ-GT) and C-reactive protein being elevated to 90 UI/L (reference range: 10-54 UI/L) and 8 mg/L (reference range: 0-0.5 mg/dl), respectively.

Stool examination for parasites, leucocytes and cysts was negative. A chest radiograph was normal. Abdominal ultrasound showed multiple hepatic lesions, dispersed in the liver parenchyma (approximately 15), oval-shaped, well-delineated, echo-poor, of variable dimensions with the largest being 25 mm. The patient was referred to a secondary care center for further evaluation and therapy.

**Figure 1:**
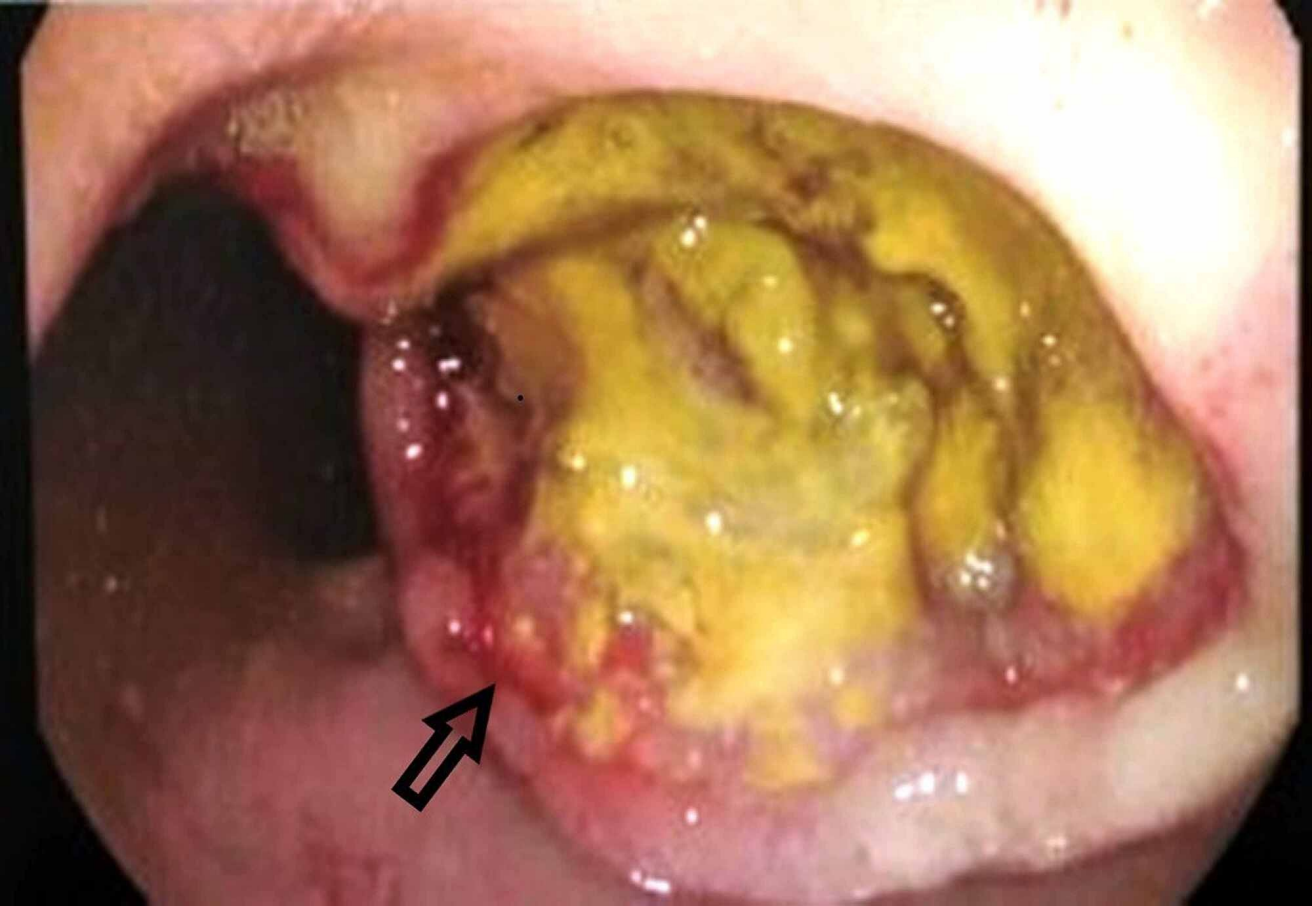
Colonoscopy showing a red elevated rectal tumor with excavation suggesting rectal cancer

The patient’s history of gastrointestinal symptoms triggered the suspicion of eosinophil‐associated end‐organ damage. Colonoscopy showed an abnormal red elevated rectal tumor with excavation suggesting rectal cancer (Figure 1, black arrow head). Histology established the diagnosis of a poorly differentiated adeno-carcinoma.

A medical oncologist and surgeon evaluated the patient. For staging, abdominal and thoracic computed tomography (CTs) were performed. Thoracic CT was clear. Abdominal CT confirmed liver metastases. Tumor biomarkers were also evaluated, and were notable for elevated carbohydrate antigen (CA 19-9) levels of 533 U/ml (normal < 37 U/ml).

He was finally diagnosed with stage IV colorectal cancer, underwent systemic preoperative chemotherapy with folinic acid, fluorouracil, oxaliplatin, and bevacizumab through an implanted central venous access port. He presented an unexpected complete clinical response to cancer treatment, and eosinophil counts returned to normal. After the primary tumor became resectable, a low anterior rectal resection was performed successfully. Three months postoperatively, he is in a good clinical state without any recurrence of neoplasia.

## Discussion

Reactive EOS occurs secondary to parasitic infections, allergies, drugs, autoimmune and neoplastic disorders [1]. EOS secondary to gastrointestinal neoplasms is a rare presentation. Research efforts by Pretlow et al. were among the first to implicate EOS in the pathophysiology of colorectal cancer (CRC) [2]. 

CRC is the most common gastrointestinal malignancy that affects people after the fifth decade of life, and for this reason screening is not recommended before 50 years of age [3]. A growing body of evidence suggests a rising incidence of CRC in younger males and mainly in the age group between 40 and 49 years [4]. Usually, it is located in the distal colon and rectum, and presents aggressive biological behavior [4-6]. Histopathological studies have shown that within this age group are more prevalent mucinous, less differentiated intestinal tumors with poor prognosis making debatable the idea to lower the screening age for CRC with preventive colonoscopy [7,8]. Late detection is usually associated with advanced stage of CRC in young patients [9].

Genetic predisposition and general lifestyle changes (lack of physical activity, dietary habits, smoking, and alcohol consumption) are well-documented risk factors that provide an explanation for the increasing incidence of CRC in the young population [6].

The differential diagnosis of EOS is broad, making the identification of its etiology challenging [1]. Primary care physicians have to be aware of the broad differential of peripheral eosinophilia in the routine clinical practice [10]. Careful history of past infections, medication exposure, residence or travel to geographic locations with parasitic endemics will provide the best evidence for individualized laboratory and imaging testing [1].

Clinical examination should assess possible upper and lower respiratory system involvement, nasal polyposis, skin rashes, lymph node enlargement, and hepato-splenomegaly [1]. Furthermore, careful systematic examination, including digital rectal examination, is important for not missing the diagnosis. When an obvious trigger for EOS is lacking, the ensuing physical examination should be targeted at the presenting symptoms and suspected organ involvement. In this direction, differential diagnosis of EOS will be performed in a cost-effective and reasonable way [1].

The role of good history taking and also the importance of clinical examination of all systems including rectal examination is crucial for not missing any clinical findings.

## Conclusions

CRC diagnosis in young patients is delayed. Patients underestimate their symptoms and physicians present a diagnostic inertia to consider the possibility of a malignant disease in differential diagnosis, especially in patients with no predisposing factors.

A timely diagnosis can drastically reduce the overall mortality and morbidity burden of the disease. Life-threatening conditions such as metastatic CRC rarely allow diagnostic revisions. Clinicians should maintain a high level of vigilance and keep in mind less frequent causes of EOS, especially when an obvious cause is missing.
